# Repurposing HIV Protease Inhibitors against West Nile Virus: Insights from Molecular Docking and Molecular Dynamics Simulations

**DOI:** 10.1021/acsphyschemau.6c00043

**Published:** 2026-06-23

**Authors:** Renato D. dos Santos, Guilherme Colherinhas, Wesley B. Cardoso

**Affiliations:** Instituto de Física, 67824 Universidade Federal de Goiás, Goiânia, Goiás 74690-900, Brazil

**Keywords:** drug repurposing, west nile virus, HIV protease inhibitors, molecular docking, molecular dynamics simulations

## Abstract

West Nile virus (WNV) is a neurotropic flavivirus lacking approved antiviral therapies. Here, we evaluated the repurposing potential of ten HIV protease inhibitors as inhibitors of the WNV NS2B–NS3 protease using molecular docking followed by molecular dynamics simulations. Structural, energetic, and interaction analyses revealed substantial differences in binding stability under dynamic conditions. Indinavir and Atazanavir consistently showed strong and stable interactions, low structural and positional fluctuations, persistent hydrogen bonding, and favorable binding free energies. In contrast, several inhibitors displayed transient binding despite favorable docking scores. These findings emphasize the importance of molecular dynamics in drug repurposing studies and identify Indinavir and Atazanavir as promising candidates for further experimental evaluation against WNV.

## Graphical abstract

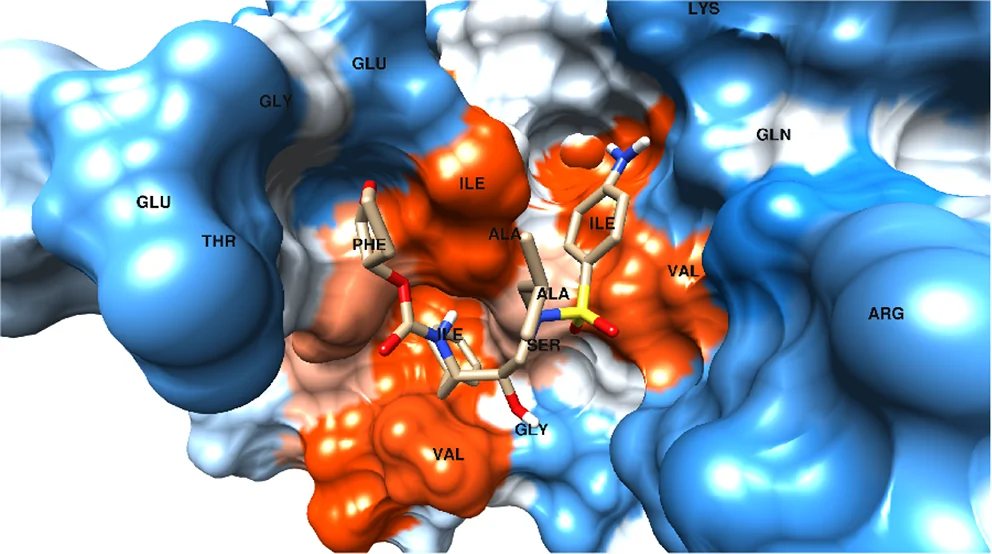

## 1 Introduction

West Nile virus (WNV) is a mosquito-borne flavivirus that belongs to the Flaviviridae family.^1^ It harbors a single-stranded, positive-sense RNA genome of approximately 11 kilobases, which encodes a single polyprotein, subsequently cleaved into both structural and nonstructural proteins. The main structural proteins include the capsid (C), premembrane (prM), and envelope (E), while nonstructural proteins, such as NS3 and NS5, play critical roles in viral replication and polyprotein processing.^2–5^

WNV enters host cells by attaching to surface receptors via its envelope protein, followed by internalization through endocytosis. Once inside the host cell, the viral RNA is released into the cytoplasm and translated into a single polyprotein, which is then processed by host and viral proteases. Replication takes place in virus-induced membrane compartments, and newly assembled virions are subsequently released to infect neighboring cells. Although WNV primarily circulates through a bird-mosquito transmission cycle, it can infect humans and other mammals, occasionally causing severe neuroinvasive diseases such as meningitis and encephalitis.^6–8^ Despite its clinical significance, no antiviral therapies have been approved to date for WNV infection.^1^

In this context, drug repurposing, also termed drug repositioning, has emerged as a promising strategy for accelerating the discovery of antiviral candidates. By exploring new indications for existing drugs with well-characterized pharmacokinetic and safety profiles, this approach significantly reduces the time and cost required in traditional drug development pipelines.^9^ Drug repurposing is particularly advantageous for combating emerging or neglected viral diseases, such as WNV, which lack targeted treatments.

Given the structural and functional similarities shared among viral proteases, HIV protease inhibitors (HPIs) have demonstrated potential off-target effects on other RNA viruses. These inhibitors, originally developed to block HIV maturation, have been extensively studied and exhibit broad-spectrum antiviral activity, including inhibition of flaviviruses such as dengue and Zika.^10–12^ This raises the possibility that HPIs may also interfere with the replication of WNV by targeting the conserved domains of its protease or other critical viral enzymes.

A recent computational study investigating the repurposing of HPIs against the SARS-CoV-2 main protease (*M* ^*pro*^) employed both molecular docking and classical molecular dynamics (MD) simulations, revealing that Nelfinavir displayed superior binding stability compared to other HPIs, such as Lopinavir and Ritonavir.^13^ These findings underscore the importance of integrating MD simulations with docking to assess the dynamic behavior of ligand–protein complexes, as static docking may overlook key conformational instabilities.

Among the essential enzymes of WNV, the NS2B–NS3 protease complex is a key molecular target for antiviral intervention. This serine protease is required for polyprotein processing and viral replication and shares mechanistic features with proteases from related viruses. Thus, it is plausible that HPIs could inhibit WNV replication by binding to and disrupting the activity of the NS2B–NS3 protease.^14^ To address this hypothesis, the present study explores the potential of repurposing clinically approved HPIs as inhibitors of the WNV NS2B–NS3 protease by using an integrated computational approach. We ask the following questions: Can HPIs effectively bind to the active site of the WNV NS2B–NS3 protease? Do molecular dynamics simulations support the stability and persistence of these interactions over time? And which inhibitors exhibit the most favorable dynamic and energetic profiles for further experimental validation?

To address these questions, this study employs a two-stage computational protocol specifically designed to investigate the inhibitory potential of clinically approved HPIs against the NS2B–NS3 protease of the West Nile virus. In the first stage, we perform molecular docking simulations to predict the binding affinities of selected HPIs and to identify key interactions within the catalytic site of the target protease. In the second stage, we carry out all-atom MD simulations in an explicit solvent to assess the stability, conformational behavior, and interaction persistence of the most promising ligand-protein complexes over time.

By combining these *in silico* techniques, the present work aims to provide a detailed molecular-level understanding of how HPIs may interfere with the function of WNV protease. Rather than relying solely on static binding predictions, our approach emphasizes the dynamic aspects of molecular recognition, which are essential for reliably identifying candidates with true inhibitory potential. The ultimate goal is to prioritize specific HPIs that exhibit favorable energetic and dynamic profiles, thus offering a rational basis for their future evaluation in *in vitro* enzymatic assays and *in vivo* models as part of antiviral development efforts against WNV.

## 2 Materials and Methods

### 2.1 Protein and Ligand Preparation

The three-dimensional crystallographic structure of the NS2B–NS3 protease complex from WNV was retrieved from the RCSB Protein Data Bank (PDB ID: 8CO8 ^15^). This structure was determined by X-ray diffraction at a resolution of 1.91 Å using the hanging-drop vapor diffusion method conducted at 293 K.^16^ All crystallographic water molecules, ligands, and nonstandard residues were removed using UCSF Chimera version 1.19 ^17^ to ensure a clean system for computational analysis. The protein structure was then processed using the DockPrep module in Chimera,^17^ which included the addition of missing hydrogen atoms, assignment of partial charges, and correction of bond orders. The resulting structure was exported in MOL2 format for subsequent molecular docking procedures. Ten clinically approved HPIs were selected for evaluation: Amprenavir, Atazanavir, Darunavir, Fosamprenavir, Indinavir, Lopinavir, Nelfinavir, Ritonavir, Saquinavir, and Tipranavir. The molecular structures of these compounds were retrieved from the PubChem database.^18^ Ligands available only in two-dimensional representation were converted into energy-minimized three-dimensional structures using Open Babel,^19^ ensuring the generation of valid conformers suitable for docking and MD simulations.

### 2.2 Molecular Docking

Molecular docking simulations were performed using AutoDock Vina version 1.2.5 ^20,21^ to predict the binding affinity and pose of each HPI with the NS2B–NS3 protease structure. A blind docking strategy was adopted by defining a search box large enough to encompass the entire protease surface, with dimensions of 53 × 58 × 47 Å^3^ and centered at coordinates (−21, 11, 20). This approach ensured an unbiased screening of all potential binding regions, allowing the detection of both orthosteric and allosteric interactions.

Although the WNV NS2B–NS3 protease possesses a well-defined catalytic site, a blind docking protocol was deliberately employed to avoid imposing *a priori* assumptions regarding the preferred binding location of structurally diverse HIV protease inhibitors. Because these compounds were originally designed against a different viral target, they may interact with regions outside the canonical active site. By extending the search space over the entire protein surface, we aimed to provide an unbiased assessment of the interaction propensity of each ligand and to identify potential alternative binding pockets that could contribute to inhibitory activity. This strategy is particularly suitable for drug repurposing studies, where noncanonical binding modes may provide additional mechanistic insights.

Docking results were ranked based on binding affinity scores (expressed in kcal/mol), and the most favorable pose for each ligand was further evaluated based on the predicted number and geometry of hydrogen bonds. A geometric criterion was applied, accepting hydrogen bonds with donor–acceptor distances *r* ≤ 3.5 Å and bond angles θ ≥ 150° (i.e., θ ≤ 30° deviation from linearity). The top-ranked docking poses were subsequently used as initial conformations for MD simulations to refine and validate binding predictions under dynamic and solvent-accessible conditions.

### 2.3 System Setup for Molecular Dynamics

The MD simulations were conducted using the GROMACS 2023.3 simulation package.^22^ The AMBER14SB force field^25–27^ was employed to model protein interactions, while the TIP3P explicit water model was used to solvate the system.^24^ Missing hydrogen atoms were added automatically based on standard protonation states at physiological pH. The NS2B–NS3 protease comprises 203 amino acid residues, with a total mass of ∼22,104 atomic mass units and a net charge of −9*e*. Ligand topologies, including atom types, partial charges, and bonded parameters compatible with the AMBER force field, were generated using Antechamber, a tool from the AMBER package,^27^ based on the prepared MOL2 files. Each protein–ligand complex was placed in the center of a rectangular simulation box measuring 7.059 × 7.601 × 6.736 nm^3^, providing at least 1.0 nm of padding between the protein and the edges of the box in all directions. This setup ensures that periodic boundary conditions are maintained without introducing artifactual interactions between periodic images of the protein. The systems were solvated with explicit water molecules, totaling approximately 10,637–10,659 molecules for the neutral system. This configuration yielded an initial solution density of approximately 995 g/L, closely resembling that of physiological aqueous environments.

We emphasize that, throughout this work, distances associated with molecular docking analyses are reported in Å, whereas structural descriptors obtained from molecular dynamics simulations are expressed in nm, in accordance with the conventional units adopted for each methodology.

### 2.4 Ion Addition and Salinity Adjustment

To mimic physiological ionic strength and maintain charge neutrality, each solvated system was supplemented with sodium (Na^+^) and chloride (Cl^–^) ions to reach a final NaCl concentration of 0.154 mol/L (corresponding to 0.9% saline). Ion addition was performed by random replacement of water molecules, with the exact number of replaced molecules varying slightly depending on the size and charge distribution of the ligand in each complex. For all analyzed systems, 77 water molecules were substituted by 43 Na^+^ and 34 Cl^–^ ions. These values were determined to maintain electroneutrality and replicate isotonic conditions, thereby supporting the structural integrity and realistic behavior of biomolecular interactions during the simulation trajectories.^23,32^

### 2.5 Energy Minimization and Equilibration

All molecular dynamics simulations followed a standardized preparation protocol consisting of energy minimization and multistep equilibration. Initially, energy minimization was performed using the steepest descent algorithm under the Verlet cutoff scheme implemented in GROMACS.^22^ A cutoff of 1.2 nm (12.0 Å) was applied for van der Waals interactions, with a smoothing function introduced between 1.0 and 1.2 nm to ensure continuity of the potential energy surface. Electrostatic interactions were calculated using the Particle Mesh Ewald (PME) method,^29^ which allows accurate treatment of long-range Coulomb interactions through a Fourier-space grid approach. This minimization phase aimed to eliminate steric clashes and unfavorable contacts, particularly at the protein–ligand and solute–solvent interfaces, thus establishing a physically stable starting structure with properly relaxed solvent geometry. Following energy minimization, equilibration of the solvated and ionized systems was carried out in a multistage protocol under periodic boundary conditions. The first equilibration stage involved a 100 ps simulation in the canonical ensemble (NVT), maintaining the number of particles, volume, and temperature constant at 310 K, a temperature representative of physiological conditions. Temperature control was achieved using a modified Berendsen thermostat^30^ with a coupling time constant (τ_*T*_) of 0.1 ps. Positional restraints were applied to all heavy atoms of the protein and the ligand to preserve the initial conformation while allowing solvent molecules and ions to equilibrate around the solute. The equations of motion were integrated using the leapfrog algorithm^33^ with a time step of 2 fs. Subsequently, a 100 ps simulation in the isothermal–isobaric ensemble (NPT) was performed to allow relaxation of the system volume and stabilization of the pressure at 1 bar. Pressure coupling was implemented via the Berendsen barostat^30^ using an isotropic pressure coupling scheme and a coupling constant (τ_*P*_) of 2.0 ps. Temperature control and positional restraints were maintained as in the NVT simulation phases. This NVT → NPT sequence was alternated iteratively to ensure full thermal and mechanical equilibrium of the system. The equilibration protocol was extended to a cumulative time of 2 ns, with alternating 100 ps NVT and NPT intervals, during which thermodynamic observables such as temperature, pressure, total energy, and density were continuously monitored to assess convergence. Bond constraints involving hydrogen atoms were applied using the LINCS (Linear Constraint Solver) algorithm,^34^ allowing the use of larger integration time steps without compromising numerical stability. Throughout equilibration, center-of-mass (COM) motion was removed to avoid artificial drift, and periodic boundary conditions were applied in all directions.

### 2.6 Production Molecular Dynamics

Upon completion of the equilibration protocol and verification of system stability, all positional restraints were removed, and a production MD simulation was initiated under NPT conditions. The production phase was conducted for 50 ns at a constant temperature (310 K) and pressure (1 bar), maintaining the same thermostat and barostat parameters used during equilibration. The time step was reduced to 1 fs in this phase to ensure improved resolution of ligand-protein interactions and enhanced numerical accuracy. Snapshots of the system were recorded every 1 ps, yielding a total of 50 000 frames per trajectory, which were used for subsequent structural, energetic, and interaction analyses. These included the assessment of ligand stability within the binding site, hydrogen bond persistence, root-mean-square deviation (RMSD), root-mean-square fluctuation (RMSF), radius of gyration (Rg), and free-energy calculations.

In addition to the hydrogen-bond population analysis, the autocorrelation function of the protein–ligand hydrogen bonds was computed using the -ac option implemented in the GROMACS gmx hbond module. The decay profile of this autocorrelation function provides information about the average lifetime and kinetic stability of the hydrogen-bond network. The corresponding free energy (Δ*G*) was estimated from the hydrogen-bond kinetics and employed as an additional descriptor of the persistence of the protein–ligand interactions throughout the MD simulations.

The simulation workflow adopted ensures a comprehensive and reproducible description of the protein–ligand dynamics in a realistic aqueous environment, providing the necessary statistical robustness for comparative analysis across all studied HIV protease inhibitors.

## 3 Analysis

The Lipinski’s Rule of Five and the Ghose Filter are empirical criteria widely used in the early screening of drug candidates, particularly in the context of oral drug development.^31,35^ In this work, the pharmacokinetic properties and drug-likeness of the selected compounds are rigorously evaluated by assessing key physicochemical parameters derived from both criteria. Specifically, properties such as molecular weight (MW), log *P* (octanol–water partition coefficient), number of hydrogen bond donors/acceptors, and molar refractivity (MR) are utilized as predictive metrics to assess the compounds’ potential for favorable ADMET (Absorption, Distribution, Metabolism, Excretion, and Toxicity) profiles, thereby refining the selection process for lead optimization. According to these guidelines, compounds with a higher likelihood of being well absorbed orally should meet at least two of the following criteria: (i) molecular weight ≤ 500 g/mol; (ii) logarithm of the octanol–water partition coefficient (log *P*) ≤ 5; (iii) number of hydrogen bond donors ≤ 5; (iv) number of hydrogen bond acceptors ≤ 10; and (v) molar refractivity between 40 and 130. Although originally designed for medicinal chemistry screenings, this rule remains a reliable parameter for assessing the “drug-likeness” of molecules in computational studies, including virtual screenings and MD simulations.

Thus, it is essential to employ complementary approaches to more comprehensively evaluate the dynamic behavior and stability of the ligand-protein complexes throughout MD simulations. In this context, one of the first steps involves assessing the energetic contributions of the system, with particular attention to van der Waals and electrostatic interactions. The Lennard-Jones and Coulomb energy components are calculated for the complex, allowing us to infer the overall stability of the system and the robustness of the interactions between the ligands and the biological target. Consistent and energetically favorable values for these interactions suggest stable and potentially effective complexes from a pharmacological perspective.

Additionally, the conformational stability of the complexes during the simulations will be evaluated through the analysis of the root-mean-square deviation (RMSD). This metric provides a quantitative estimate of structural fluctuations over time by measuring the displacement of the atomic positions relative to a reference structure. By analyzing the RMSD of different regions of the system, such as the protein backbone and the ligand, it is possible to determine whether significant conformational changes occur or whether the structure remains stable. Small and consistent fluctuations indicate that the complex maintains its structural integrity throughout the simulation, whereas pronounced deviations may point to instability or structural reorganization.

Other structural parameters will also be investigated, such as the radius of gyration (Rg), which reflects the compactness of the protein structure. Variations in Rg may indicate folding, relaxation, or even unfolding events induced by ligand binding. This analysis will be complemented by the monitoring of specific interatomic distances to identify key interactions at the binding site, as well as by the quantification of hydrogen bonds formed between the protein and the ligand throughout the simulation trajectory. The persistence and stability of these hydrogen bonds are indicative of key interactions that may significantly contribute to binding affinity and specificity. A detailed discussion of these properties is presented in the following section, aiming to integrate structural and energetic data into the overall assessment of the potential efficacy of the selected ligands.

## 4 Results and Discussion

### 4.1 Molecular Docking

Beyond the initial evaluation based on Lipinski’s Rule of Five, the molecular docking analysis provided additional insights into the binding potential of the selected antiviral compounds with the WNV protease (PDB ID: 8CO8). Based on the data provided in Table 1, the molecular docking analysis revealed that all 10 HIV protease inhibitors exhibited favorable binding affinities toward the WNV protease active site, with the predicted free energy of binding ranging from the strongest interaction of −9.1 kcal/mol observed for Saquinavir to the weakest, yet still significant, interaction of −7.1 kcal/mol for Amprenavir. This predictive in silico potency suggests that these compounds are structurally capable of establishing stabilizing interactions within the target pocket. However, a comprehensive assessment of their drug-likeness profile, based on widely recognized empirical criteria for oral drug candidates, indicates significant liabilities across the entire panel. An evaluation againstLipinski’s Rule and the Ghose Filter parameters demonstrated near-universal noncompliance, particularly concerning molecular size and volume. All 10 compounds strictly violate the criteria for molecular weight (MW ≤ 500 g/mol) and molar refractivity (MR range 40–130), with MW values ranging from 505.6 g/mol (Amprenavir) up to 720.9 g/mol (Ritonavir) and MR values exceeding 130 across the board. These findings indicate that all candidate molecules are excessively large and voluminous for optimal passive diffusion across biological membranes. Furthermore, six out of the ten compounds also violate the criterion for the octanol–water partition coefficient (log *P* ≤ 5), with values extending up to 7 for Tipranavir, signaling potentially high lipophilicity, which may compromise aqueous solubility and increase the risk of off-target binding. In summary, the collective data highlight that while these compounds exhibit excellent in silico binding affinities toward the WNV protease, their physicochemical profiles indicate multiple violations of Lipinski’s and Ghose criteria. These molecules are clinically validated HIV protease inhibitors, demonstrating that their therapeutic efficacy is achieved, often through advanced formulation or coadministration strategies to circumvent pharmacokinetic hurdles. Therefore, the primary goal of this work shifts from initial drug-likeness screening to utilizing these potent scaffolds to understand, via MD simulations, the precise effects on the stability of the binding interactions and their inhibitory mechanism against the WNV protease.

**Table 1:** Molecular Docking Analysis of Several Compounds against the West Nile Virus Protease and Properties of Potential Inhibitor candidates^18,28^

PubChem CID	Name	Affinity (kcal*/*mol)	Molecular weight (≤500 g/mol)	Log *P* (≤5)	H-Bond donor (≤5)	H-bond acceptor (≤10)	Molar Refractivity (40–130)
441243	Saquinavir	–9.1	670.8	4.2	5	7	192.87
54682461	Tipranavir	–8.6	602.7	7	2	10	153.80
92727	Lopinavir	–8.5	628.8	5.9	4	5	187.92
5362440	Indinavir	–8.4	613.8	2.8	4	7	182.62
392622	Ritonavir	–8.3	720.9	6	4	9	197.82
64143	Nelfinavir	–8.2	567.8	5.7	4	6	166.17
148192	Atazanavir	–8.0	704.9	5.6	5	9	193.76
213039	Darunavir	–7.6	547.7	2.9	3	9	142.20
131536	Fosamprenavir	–7.5	585.6	1.8	4	11	144.53
65016	Amprenavir	–7.1	505.6	2.9	3	8	133.62

As an example, in Figure 1 we illustrate the binding mode of Amprenavir within the active site of the WNV NS2B–NS3 protease, highlighting the spatial relationship between the ligand and the surrounding catalytic residues. The representation emphasizes the proximity of Amprenavir to key amino acids that define the protease pocket, providing a structural context for the interactions predicted by molecular docking. The orientation of the ligand within this region suggests favorable shape complementarity with the active-site cavity and potential polar and hydrophobic contacts with neighboring residues, which are essential for molecular recognition. This structural snapshot serves as a qualitative reference for the subsequent molecular dynamics analyses, linking the static docking pose to the energetic and dynamic behavior observed during the simulations.

**Figure 1:**
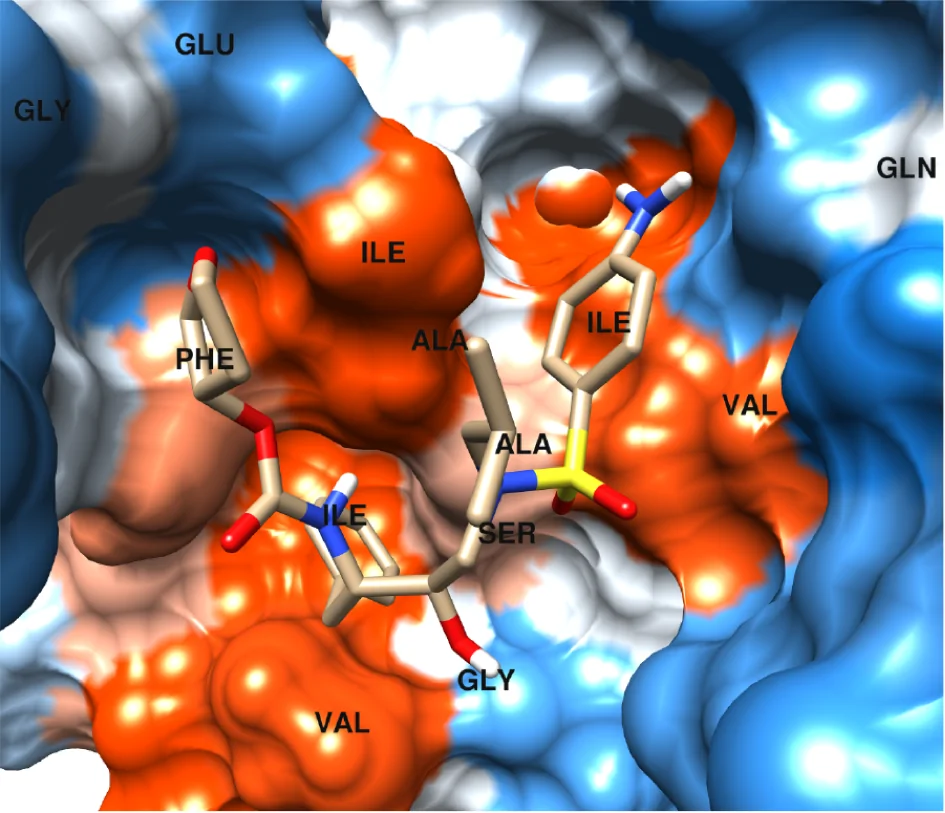
Active-site representation of the WNV NS2B–NS3 protease in complex with Amprenavir, highlighting neighboring residues involved in ligand binding.

### 4.2 Molecular Dynamics Simulations

To complement the docking results and assess the stability and nature of protease-inhibitor interactions over time, MD simulations were conducted by considering the top-ranked docking pose, using consistent force fields and simulation parameters to ensure a uniform and comparable analysis across all systems.

The results summarized in Figure 2 reveal clear differences in the energetic driving forces and dynamic stability of the HIV protease inhibitors bound to the WNV NS2B–NS3 protease. Indeed, Atazanavir and Indinavir emerge as the most energetically robust complexes, combining highly favorable total interaction energies (−310.2 and −288.8 kJ/mol, respectively) with comparatively low total energy fluctuations (9.0% and 12.4%, respectively). This limited variability indicates that their strong binding is maintained consistently over the simulation time, reflecting a stable interaction network supported by both electrostatic and van der Waals contributions. Ritonavir shows a similar trend, with a favorable total interaction energy (−267.2 kJ/mol) and moderate total fluctuation (12.1%), suggesting sustained, albeit slightly more flexible, binding. In contrast, Amprenavir, Fosamprenavir, Lopinavir, Nelfinavir, Saquinavir, and Tipranavir display markedly higher fluctuations in the Coulombic component (≈63–122%), indicating pronounced variability in electrostatic contacts, likely arising from transient rearrangements of charged and polar groups within the binding pocket. Although their Lennard-Jones interactions are comparatively more stable (generally <20% fluctuation), the large Coul-SR variability propagates into higher total interaction energy fluctuations (≈21–25%), consistent with less persistent binding. Darunavir represents an intermediate case, with moderate total stabilization (−230.3 kJ/mol) and intermediate fluctuation (17.6%), reflecting partial but unstable engagement with the protease. Importantly, these energetic trends correlate with the observed structural fluctuations: ligands characterized by low total energy variability, particularly Atazanavir and Indinavir, exhibit more restrained conformational fluctuations of the complex, whereas compounds with large energetic variability show enhanced structural fluctuations, indicative of intermittent binding and reduced dynamic stability. Overall, this analysis underscores that not only the magnitude of the interaction energy but also its temporal stability is critical for discriminating the most promising inhibitors, reinforcing Indinavir and Atazanavir as leading candidates based on their favorable balance between binding strength and low fluctuation.

**Figure 2:**
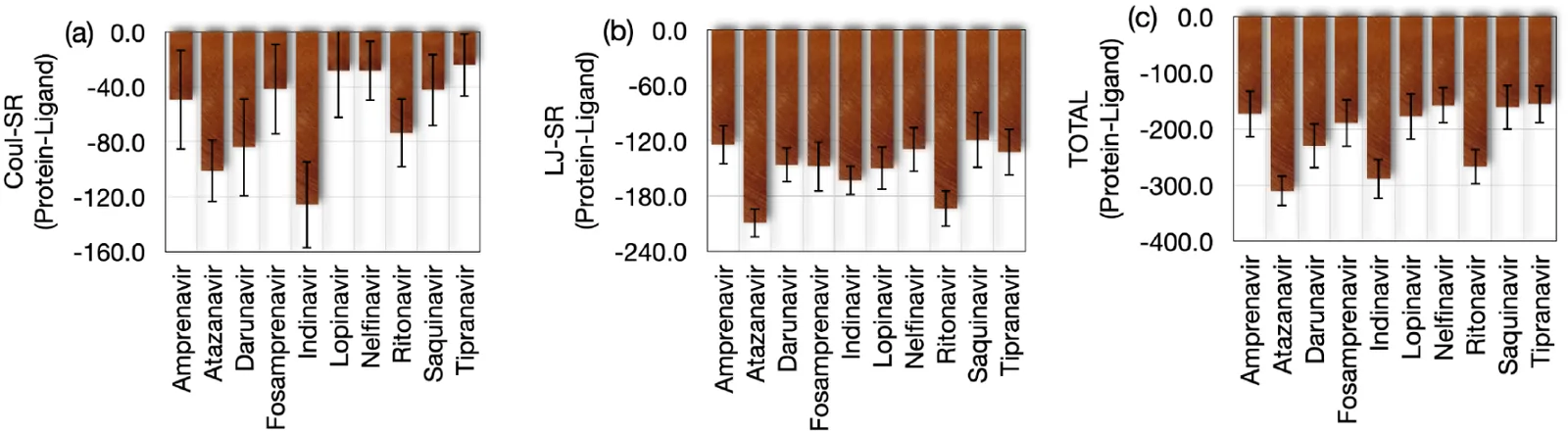
Short-range (SR) protein–ligand interaction energy components obtained from 50 ns MD simulations of the WNV NS2B–NS3 protease complexes. Panels show the time-averaged contributions of: (a) Coulombic short-range (SR) electrostatic interactions (Coul-SR), (b) Lennard-Jones short-range (SR) van der Waals interactions (LJ-SR), and (c) the total protein–ligand interaction energy, calculated as the sum of Coul-SR and LJ-SR terms. Energies were extracted over the equilibrated portion of the trajectories and reflect the relative stability and strength of polar and nonpolar interactions governing ligand binding within the protease active site.

The results presented in Figure 3 provide complementary insight into the global structural stability of the protease and the dynamic behavior of ligand binding. The RMSD-*C*_α_ values remain low for all systems (≈0.17–0.25 nm), indicating that none of the inhibitors induce significant backbone rearrangements of the WNV NS2B–NS3 protease. Lopinavir, Amprenavir, and Darunavir exhibit particularly stable backbone behavior, with moderate *C*_α_ fluctuations (<15%), whereas Atazanavir and Nelfinavir show larger relative backbone fluctuations, suggesting increased local flexibility despite the preserved global fold. In contrast, the protein–ligand RMSD reveals pronounced differences in binding stability. Indinavir stands out with the lowest average RMSD (0.20 nm) and limited fluctuation (24.4%), consistent with persistent and well-anchored binding throughout the simulation. Atazanavir, Darunavir, and Ritonavir display intermediate values, indicative of stable but more adaptable binding modes. Conversely, Amprenavir, Fosamprenavir, Lopinavir, Saquinavir, and Tipranavir show elevated protein–ligand RMSD values and large fluctuations (≈40–63%), reflecting substantial positional variability and partial disengagement from the binding pocket. The radius of gyration remains narrowly distributed across all complexes (≈1.69–1.73 nm), with minimal fluctuations, confirming that ligand binding does not compromise the overall compactness of the protease. Collectively, these results indicate that while the global protein structure is preserved for all systems, only a subset of inhibitors (most notably Indinavir) achieve a favorable balance between low ligand positional variability and minimal perturbation of the protease scaffold, reinforcing its superior dynamic stability relative to the remaining compounds.

**Figure 3:**
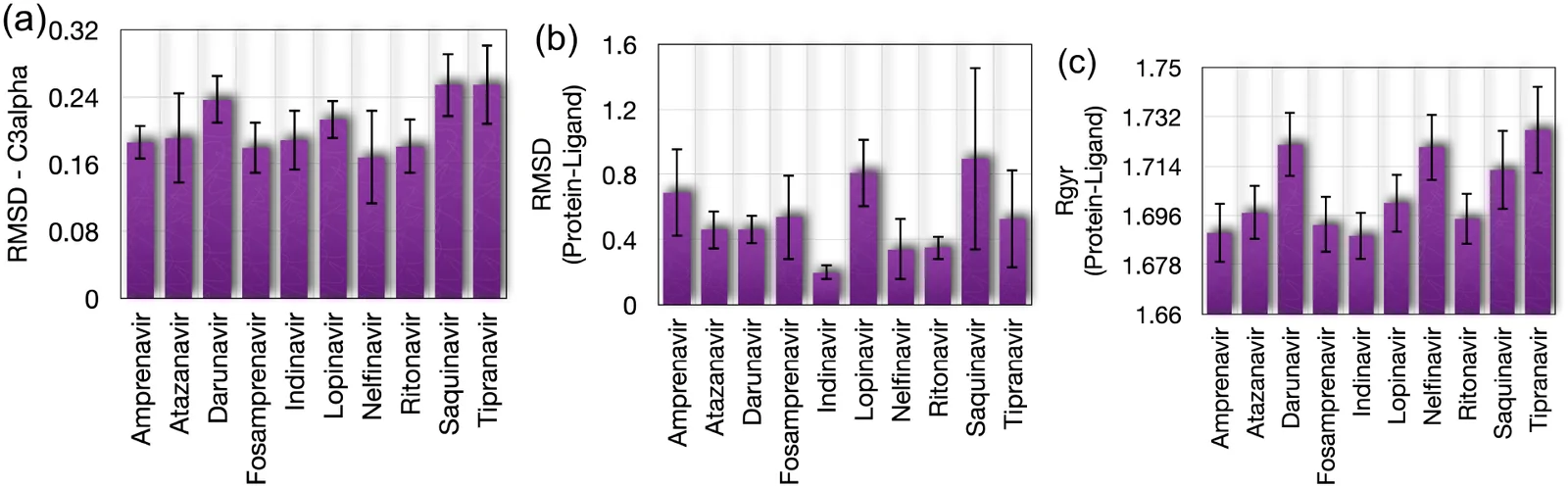
Time-averaged structural descriptors obtained from 50 ns MD simulations of the WNV NS2B–NS3 protease complexes with HIV protease inhibitors: (a) *C*_α_ RMSD of the protein backbone, (b) protein–ligand RMSD, and (c) radius of gyration (Rg) of the protein–ligand complex. Bars represent mean values calculated over the equilibrated portion of the trajectories, while error bars correspond to the statistical fluctuations (standard deviation) of each metric, as reported in the associated table.

The center-of-mass distance analysis shown in Figure 4 offers a direct metric of ligand retention within the protease binding pocket throughout the simulations. Indinavir and Atazanavir exhibit the smallest average center-of-mass (COM) distances (1.72 and 1.85 nm, respectively) together with minimal fluctuations (∼2%), indicating that these ligands remain tightly localized near the binding site with highly stable positional behavior. Fosamprenavir and Ritonavir also display relatively short COM distances (≈1.72–1.78 nm) and low variability (<6%), suggesting sustained engagement with the protease despite modest internal flexibility. In contrast, Darunavir, Nelfinavir, and Tipranavir show the largest COM distances (>2.18 nm), consistent with a more peripheral or partially displaced binding mode, even though their relative fluctuations remain moderate. Amprenavir and Saquinavir combine intermediate-to-large COM distances with pronounced fluctuations (10–12%), reflecting intermittent positional instability and episodes of ligand drift away from the binding pocket.

**Figure 4:**
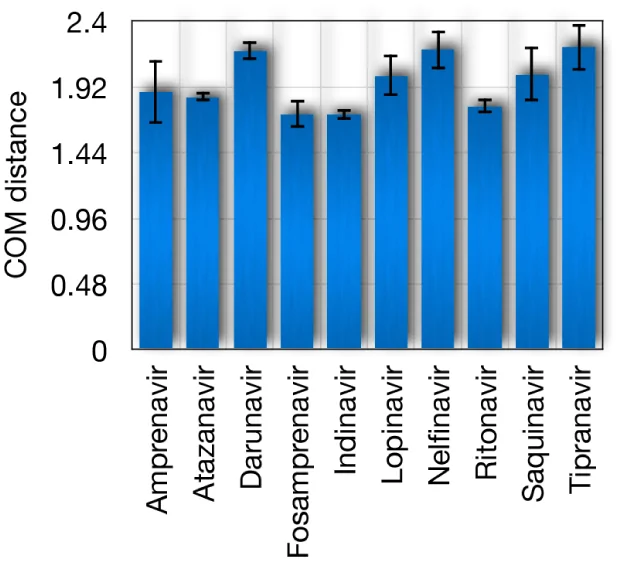
Time-averaged center-of-mass (COM) distance between the WNV NS2B–NS3 protease and the bound ligand obtained from 50 ns MD simulations. Error bars represent the statistical fluctuation (standard deviation) of the COM distance over the trajectory, providing a quantitative measure of ligand positional stability relative to the protease binding site.

Although the average COM distances differ only slightly among the investigated systems, the associated temporal fluctuations provide additional information regarding the ligand’s positional stability. In this context, the comparatively lower fluctuation values observed for atazanavir and indinavir support their enhanced stability within the protease binding pocket during the MD simulations.

The results in Figure 5 highlight marked differences in both the specificity and strength of ligand–protease interactions. Atazanavir exhibits the highest average number of hydrogen bonds (3.09) combined with low fluctuation (22.5%), indicating persistent and well-defined polar interactions within the binding site, which is accompanied by a comparatively low free-energy value (Δ*G* = 10.5 kJ/mol) obtained from the hydrogen-bond autocorrelation analysis. Ritonavir and Indinavir also show relatively high hydrogen bond counts (∼1.8–1.9) with limited variability, suggesting stable hydrogen-bond networks that are accompanied by intermediate free-energy values (Δ*G* ≈ 20 kJ/mol). In contrast, Amprenavir, Fosamprenavir, Lopinavir, Nelfinavir, Saquinavir, and Tipranavir display low average hydrogen bond numbers (<1.1) accompanied by very large fluctuations (≈90–200%), reflecting transient and poorly sustained polar contacts. These ligands also exhibit comparatively larger free-energy values (Δ*G* ≈ 18–27 kJ/mol), indicating weaker overall stabilization. Darunavir occupies an intermediate regime, forming a moderate number of hydrogen bonds (1.88) but with substantial variability, which is mirrored by its intermediate Δ*G* value (21.4 kJ/mol). Collectively, the hydrogen-bond and free-energy analyses provide complementary descriptors of the protein–ligand interaction patterns. Notably, Atazanavir exhibits the highest hydrogen-bond occupancy and one of the lowest relative fluctuations among the investigated systems, whereas Indinavir and Ritonavir also present relatively persistent hydrogen-bond networks that support their overall stable interaction profiles.

**Figure 5:**
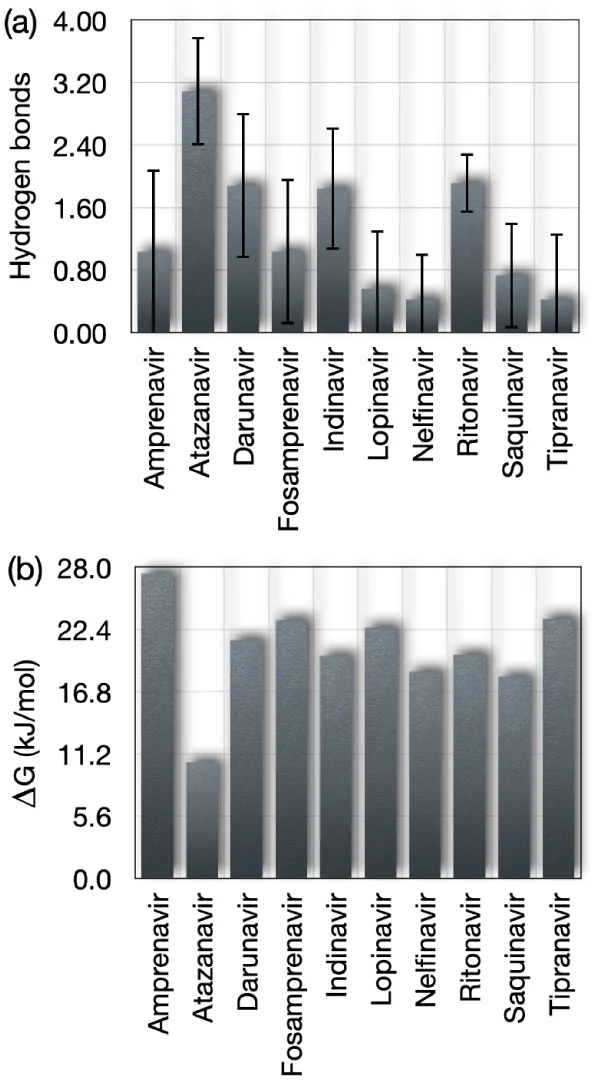
Panel (a) reports the mean protein–ligand hydrogen bond count averaged over all sampled configurations, with error bars representing the statistical fluctuation (standard deviation). Panel (b) shows the estimated free-energy values (Δ*G*, kJ mol^–1^) obtained from the hydrogen-bond autocorrelation analysis.

In addition to the global stability descriptors, the interaction profiles of the top-performing ligands provide further mechanistic insight into their potential inhibitory activity. Although the most stable binding poses do not involve direct hydrogen-bond formation with the catalytic triad residues, persistent interactions are established with residues located within the substrate-binding region of the NS2B–NS3 protease, including Glu148, Asn180, Ala192, Ile193, and Gln195. These observations suggest that the favorable energetic and structural profiles of the leading compounds arise from their ability to maintain a stable interaction network within the conserved binding pocket, which may interfere with substrate accommodation and enzymatic function.

Taken together, the structural, energetic, and interaction analyses provide a coherent and self-consistent picture of the binding behavior of HIV protease inhibitors to the WNV NS2B–NS3 protease. While all compounds preserve the global structural integrity of the protease, as evidenced by stable backbone deviations and nearly constant radii of gyration, pronounced differences emerge in ligand stability and binding quality. Indinavir and Atazanavir consistently combine favorable interaction energies, low positional and conformational fluctuations, short and stable center-of-mass distances, and well-maintained hydrogen-bond networks, indicating persistent and well-anchored binding within the active site. Ritonavir and Fosamprenavir display intermediate behavior, with reasonable energetic stabilization but increased variability in ligand positioning or polar contacts, suggesting more flexible binding modes. In contrast, Amprenavir, Lopinavir, Saquinavir, Tipranavir, and Nelfinavir are characterized by large energetic and structural fluctuations, elevated ligand mobility, weak and highly variable hydrogen bonding, and less favorable free energies, all of which point to transient or unstable interactions. Collectively, these results demonstrate that strong average interaction energies alone are insufficient to ensure effective binding; rather, the combination of energetic favorability with low dynamic fluctuation and persistent intermolecular contacts is essential. On this basis, Indinavir and Atazanavir emerge as the most robust candidates, exhibiting the most balanced and stable interaction profiles among the evaluated inhibitors, and therefore represent the most promising scaffolds for further experimental validation against the WNV protease.

## 5 Conclusion

In this work, an integrated computational framework combining molecular docking and extensive MD simulations was employed to evaluate the potential of clinically approved HIV protease inhibitors as repurposed inhibitors of the West Nile virus (WNV) NS2B–NS3 protease. While docking results indicated that all evaluated compounds are capable of occupying the protease active site, the subsequent dynamic analyses revealed substantial differences in binding stability, interaction persistence, and energetic robustness that cannot be captured by static approaches alone.

Across all systems, the global fold and compactness of the WNV protease were preserved, indicating that ligand binding did not induce deleterious structural perturbations. However, ligand-dependent effects were clearly observed at the local level. Indinavir and Atazanavir consistently demonstrated the most favorable balance among strong interaction energies, low structural and positional fluctuations, short and stable center-of-mass distances, and persistent hydrogen-bond networks. These features collectively point to stable and well-defined binding modes that are maintained throughout the simulation time, highlighting their superior dynamic compatibility with the WNV protease active site. Ritonavir and Fosamprenavir exhibited intermediate behavior, characterized by reasonable energetic stabilization but increased flexibility in ligand positioning or intermolecular contacts.

In contrast, Amprenavir, Lopinavir, Saquinavir, Tipranavir, and Nelfinavir showed pronounced energetic and structural fluctuations, elevated ligand mobility, and weak or highly variable hydrogen-bonding patterns, suggesting transient binding and reduced inhibitory potential under dynamic conditions. These findings underscore the limitations of relying exclusively on docking scores and emphasize the critical role of MD simulations in discriminating truly stable binders from those that are only statically favorable.

Overall, this study identifies indinavir and atazanavir as the most promising candidates for repurposing against WNV, based on a comprehensive assessment of energetic, structural, and interaction-based descriptors. Beyond the specific compounds evaluated, the present work reinforces the importance of integrating dynamic and thermodynamic analyses into antiviral drug repurposing pipelines, particularly for neglected and emerging viral pathogens, such as West Nile virus. Experimental validation of the highlighted candidates will be essential to confirm their inhibitory activity and further explore their potential as leads for antiviral development.
